# Identification of four novel QTL linked to the metabolic syndrome in the Berlin Fat Mouse

**DOI:** 10.1038/s41366-021-00991-3

**Published:** 2021-10-23

**Authors:** Manuel Delpero, Danny Arends, Maximilian Sprechert, Florian Krause, Oliver Kluth, Annette Schürmann, Gudrun A. Brockmann, Deike Hesse

**Affiliations:** 1 Albrecht Daniel Thaer-Institut für Agrar- und Gartenbauwissenschaften, Humboldt-Universität zu Berlin, Berlin, Germany; 2 Department für Experimentelle Diabetologie, Deutsches Institut für Ernährungsforschung Potsdam-Rehbrücke (DIfE), Nuthetal, Germany; 3 German Center for Diabetes Research (DZD), München-Neuherberg, Germany; 4 University of Potsdam, Institute of Nutritional Science, Potsdam, Germany

**Keywords:** Genetics, Obesity, Type 2 diabetes

## Abstract

**Background:**

The Berlin Fat Mouse Inbred line (BFMI) is a model for obesity and the metabolic syndrome. This study aimed to identify genetic variants associated with impaired glucose metabolism using the obese lines BFMI861-S1 and BFMI861-S2, which are genetically closely related, but differ in several traits. BFMI861-S1 is insulin resistant and stores ectopic fat in the liver, whereas BFMI861-S2 is insulin sensitive.

**Methods:**

In generation 10, 397 males of an advanced intercross line (AIL) BFMI861-S1 × BFMI861-S2 were challenged with a high-fat, high-carbohydrate diet and phenotyped over 25 weeks. QTL-analysis was performed after selective genotyping of 200 mice using the GigaMUGA Genotyping Array. Additional 197 males were genotyped for 7 top SNPs in QTL regions. For the prioritization of positional candidate genes whole genome sequencing and gene expression data of the parental lines were used.

**Results:**

Overlapping QTL for gonadal adipose tissue weight and blood glucose concentration were detected on chromosome (Chr) 3 (95.8–100.1 Mb), and for gonadal adipose tissue weight, liver weight, and blood glucose concentration on Chr 17 (9.5–26.1 Mb). Causal modeling suggested for Chr 3-QTL direct effects on adipose tissue weight, but indirect effects on blood glucose concentration. Direct effects on adipose tissue weight, liver weight, and blood glucose concentration were suggested for Chr 17-QTL. Prioritized positional candidate genes for the identified QTL were *Notch2* and *Fmo5* (Chr 3) and *Plg* and *Acat2* (Chr 17). Two additional QTL were detected for gonadal adipose tissue weight on Chr 15 (67.9–74.6 Mb) and for body weight on Chr 16 (3.9–21.4 Mb).

**Conclusions:**

QTL mapping together with a detailed prioritization approach allowed us to identify candidate genes associated with traits of the metabolic syndrome. In addition, we provided evidence for direct and indirect genetic effects on blood glucose concentration in the insulin-resistant mouse line BFMI861-S1.

## Introduction

The metabolic syndrome is defined as a metabolic abnormality that leads to high body weight, ectopic fat storage, insulin resistance, high blood pressure, and chronic low-grade inflammation [1]. Heritability estimates for each trait of the metabolic syndrome are high with some estimates exceeding 50% [2]. Nevertheless, genome-wide association studies on body mass index and other traits of the metabolic syndrome identified loci, that combined, account for only 1–7% of the variance in the examined population [2]. Therefore, studies on different populations are needed to identify additional causal genes to better understand their direct and interaction effects contributing to the metabolic syndrome.

The goal of the current study was to identify genetic factors contributing to obesity and glucose homeostasis in the Berlin Fat Mouse. Originally, the Berlin Fat Mouse population was selected for juvenile obesity. After 58 generations of selection, different Berlin Fat Mouse Inbred (BFMI) lines were generated through repeated brother–sister mating [3]. In a cross between the most obese inbred line BFMI860 and the lean control line C57BL/6NCrl, we have previously identified a recessive genetic defect at a locus on chromosome (Chr) 3 accounting for 40% of the variance in adipose tissue weight at 6 weeks [4, 5]. This juvenile obesity locus (*jObes1*) is fixed in all BFMI sublines.

In the current study, we used the inbred lines BFMI861-S1 (S1) and BFMI861-S2 (S2). S1 and S2 are sublines created from the BFMI860, as such the BFMI860 is the predecessor of the S1 and S2. The S1 and S2 lines were conspicuously different with respect to metabolic traits [6]. In particular, the S1 line showed high body weight, hepatic fat storage, low insulin sensitivity, and impaired glucose tolerance. In contrast, S2 is insulin sensitive despite being obese [6]. This observation was particularly interesting since these two lines were derived from one parental line that was divided into two sub-lines only after four generations of inbreeding. Therefore, these two lines are genetically highly similar, and the remaining genetic diversity is responsible for phenotypic differences. To identify genetic loci accounting for the observed obesity, and glucose homeostasis in S1 mice, we performed a quantitative trait locus (QTL) mapping study in an advanced intercross line (AIL) which was generated from an initial cross between the BFMI861 lines S1 and S2. In this study, all AIL mice were challenged with a high-fat, high-carbohydrate diet.

## Material and methods

### Mouse population

We used male mice of the parental mouse lines BFMI861-S1 (S1) and BFMI861-S2 (S2) and generation 10 of an AIL population. The AIL population was generated from an initial cross between a BFMI861-S1 (S1) male and a BFMI861-S2 (S2) female followed by repeated random mating in every generation. For randomization of mating pairs, the program RandoMate [7] was used. The BFMI861 lines S1 and S2 were generated as described in Heise et al. [6].

### Animal husbandry

All experimental treatments of mice were approved by the German Animal Welfare Authorities (approval no. G0235/17). Mice were kept under conventional conditions with a 12:12 h light–dark cycle (lights on at 0600 h) and at a temperature of 22 ± 2 °C. Mice had ad libitum access to food and water.

### Experiment and phenotyping

Data from parental strains S1 and S2 were collected at 20 weeks on a standard diet containing 16.7 MJ/kg of metabolizable energy, 11% from fat, 26% from protein, and 53% from carbohydrates (V1534-000, ssniff EF R/M; Ssniff Spezialdiäten GmbH, Soest, Germany) and blood glucose was measured at 25 weeks after 5 weeks exposure to a high-fat, high-carbohydrate diet containing 21.9 MJ/kg of metabolizable energy, 28% from fat, 20% from protein and 40% from carbohydrates [8].

To emphasize the difference in glucose homeostasis, all AIL animals were challenged with a dietary regime that provides a gluco-lipotoxic environment for the β-cells and thereby provokes differences in β-cell resilience [9]. This dietary regime challenge was undertaken to provoke differences in the phenotypes studied. Until the age of 20 weeks, AIL mice were fed the rodent standard diet. In weeks 21 and 22, mice were fed a high-fat, low-carbohydrate diet, containing 16.9 MJ/kg of metabolizable energy, 34% from fat, 19% from protein, and 47% from carbohydrates (C1057; Altromin Spezialfutter GmbH & Co. KG, Lage, Germany) to increase obesity but to protect β-cells. Afterward, animals were fed for 3 weeks a high-fat, high-carbohydrate diet containing 21.9 MJ/kg of metabolizable energy, 28% from fat, 20% from protein, and 40% from carbohydrates [8] to challenge β-cells with carbohydrates and thereby increase differences in glucose metabolism.

AIL mice were phenotyped between the age of 3 (after weaning) and 25 weeks. Body mass was recorded weekly. To investigate glucose metabolism, an oral glucose tolerance test (oGTT) was performed in week 18 and an intraperitoneal insulin tolerance test (ITT) in week 20 as described before [6]. The area under the curve (AUC) for blood glucose concentration of oGTT and ITT was calculated. At 25 weeks, final blood glucose concentration was recorded after fasting for two hours. Afterward, mice were anesthetized with isoflurane and sacrificed [10]. Gonadal adipose tissue (GonAT), subcutaneous adipose tissue, liver, and skeletal muscle (quadriceps) were dissected and weighed. Tissues were collected in liquid nitrogen and stored at −80 °C. Protein content and triglycerides of homogenized liver samples were determined as described in Hesse et al. [11].

Outliers, defined as individuals who have a measurement that deviates from the population mean by more than four standard deviations (SD), were removed from the data. Pearson’s correlation coefficients were calculated between normal distributed traits. For non-normal distributed traits, Spearman’s correlation coefficients were calculated.

### Genotyping

Out of the 397 males that were phenotyped, selective genotyping was performed; 200 mice representing the two tails of the phenotypic distributions of gonadal adipose tissue weight and liver weight were selected for genotyping with the Giga Mouse Universal Genotyping Array (GigaMUGA; Illumina, San Diego, CA, USA) [12]. Genotyping was done at Neogen GeneSeek (Lincoln, NE, USA). Due to high genetic similarity of the parental lines S1 and S2 of the AIL population, only 5215 out of 143,259 SNPs on the array were informative and passed the quality control (supplementary Fig. 1, supplementary File 1).

Remaining 197 males of the AIL population were genotyped for 7 top markers. For these markers, KASP genotyping assays were developed as described previously [13] (supplementary File 2). The additional animals were genotyped to counteract any bias in the estimates of allele effect sizes introduced by selective genotyping.

### QTL mapping

QTL mapping was performed in two steps: First, a QTL scan was performed using the 200 males that were genotyped with the GigaMUGA Array. Afterward, a final QTL scan was performed including all animals (genotyped by GigaMUGA and KASP).

Covariates (subfamily and litter size) were investigated for a significant influence on each phenotype. Covariate analysis showed that litter size significantly influenced liver weight (*p* < 0.02), as such, litter size was added as a covariate to the model when QTL mapping liver weight. No other significant covariates were found. Using pedigree information of the AIL population, we tested the sub-family effect on the phenotype, but no significant influence was found (code available upon request).

To confirm that QTL mapping models are valid, residuals of the models were tested for normality using a Shapiro-Wilk test. If residuals were found to not be normally distributed, a nonparametric Kruskal–Wallis one-way analysis of variance was performed to validate the top marker.

The number of independent statistical tests was estimated by simpleM [14] which determined the number of independent tests to be 849 (window size = 820, mEff = 849). Afterwards Bonferroni correction for multiple testing correction [15] was performed using the number of independent SNPs as determined by simpleM. *P* values were converted to LOD scores, using LOD = −log10(*p* value). LOD scores above 4.9 and 4.2 were deemed to be genome-wide highly significant and significant, respectively. The 95% confidence interval of a QTL was determined by a 1.5 LOD drop from the top SNP position [16]. Start and end positions were defined as the first SNP upstream or downstream of the 1.5 LOD-drop confidence interval.

### Causal modeling

In case of an overlapping QTL between multiple traits, we applied pairwise causal modeling as previously described [17, 18]. In short, when a common QTL is found for two (or more traits), we model the effect of the QTL on these traits in a pairwise manner. Causal modeling was performed by comparing the independent model (QTL directly affects both T1 and T2) with the causal/reactive model (QTL directly affects T1 which in turn affects T2). Of course, it can happen that none of the models fit the data satisfactory, we then assume causality is undetermined. Statistical models used for causal modeling are described in more detail in supplementary File 3.

Direct QTL effects are defined as caused by a QTL which directly affects the variability of both traits (independent model fits best, QTL directly affects T1 and T2). Indirect effects were defined as effects on a trait (T1) caused by a QTL through another trait (T2) (causal model fits best). In this case, the QTL is defined as having a direct effect on T2, and an indirect effect on T1. Causal modeling to determine direct and indirect effects of QTL on traits was performed for GonAT weight, liver weight, and blood glucose concentration on Gatlgq and for GonAT weight and blood glucose concentration on Gatq1.

### Whole-genome sequencing

The two parental lines of the AIL (S1 and S2) were paired-end sequenced using the “Illumina HiSeq” (Illumina) platform. Obtained DNA reads were trimmed and aligned to the mouse genome (MM10, GRCm38.p3), sequence variants were called using BCFtools and annotated using the Ensembl Variant Effect Predictor (VEP) [19, 20]. VEP provided information on the position of SNPs within known motifs such as promoters, regulatory sites, and protein domains. DNA sequencing data were deposited at the NCBI Sequence Read Archive under BioProject ID: PRJNA717237.

### Gene expression analysis

RNA was isolated from gonadal adipose tissue (S1: *n* = 7, S2: *n* = 8), liver (S1: *n* = 7, S2: *n* = 8) and skeletal muscle (S1: *n* = 7, S2: *n* = 8) of males of the parental lines S1 and S2 at 10 weeks. Pancreatic islets (S1: *n* = 6, S2: *n* = 6) were isolated as described in Gotoh et al. [21] and RNA was extracted as described [10]. Gene expression was measured with the Clariom S assay for mouse (Thermo Fisher Scientific) using service (ATLAS Biolabs, Berlin, Germany). The intensity values of the arrays were transformed to the logarithm of base 2 and quantile normalized for each tissue separately. To test for expression differences between S1 and S2 mice, t-tests were performed for each probe on the array in each tissue. Benjamini-Hochberg correction was applied for multiple testing. R was used for statistical analysis and graphical presentation [22]. Quantitative real-time PCR was performed as described in Heise et al. [6]. Relative transcript amounts were calculated using the relative quantification method (ddCT-method) [23]. Gene-specific primers are available in supplementary File 4.

### Candidate gene prioritization

Genomic DNA sequences of all protein-coding positional candidate genes were downloaded using bioMART [24]. To include regulatory regions such as promoters, we considered additional 1000 base pairs from the start and end position of each gene. Monomorphic genes without sequence variants between S1 and S2 were removed from the list of positional candidate genes. All other genes were scored for potential functional effects of sequence variants, gene expression differences between S1 and S2 in gonadal adipose tissue and liver, and their contribution to KEGG pathways [25]. Coding sequence variants leading to stop gain/stop loss codons and missense mutations located in functional protein domains were awarded 3 points to the gene score. A missense variant with either a deleterious or a tolerated SIFT (Sorting Intolerant From Tolerant) value obtained 3 or 1 point, respectively. Non-coding variants were scored based on their location in potential functional sites. If a non-coding variant was located in the promoter or in a splice site, 3 points were awarded; if located in untranslated regions (UTRs), enhancers, or CTCF binding sites (involved in 3D structure of chromatin) 1 point was awarded. Genes differentially expressed in at least one tissue were awarded 2 points. Genes annotated in relevant KEGG metabolic pathways were awarded 1 point. Genes in KEGG pathways were downloaded using the R package “StarBioTrek” [26]. To find further evidence for potential causality, the highest scored candidate genes were screened for metabolic processes or diseases using Gene Ontology, public literature, and databases such as Mouse Genome Informatics and the International Mouse Phenotyping Consortium.

## Results

### Response of parental lines S1 and S2, and AIL males to high-fat, high-carbohydrate diet

**Fig. 1:**
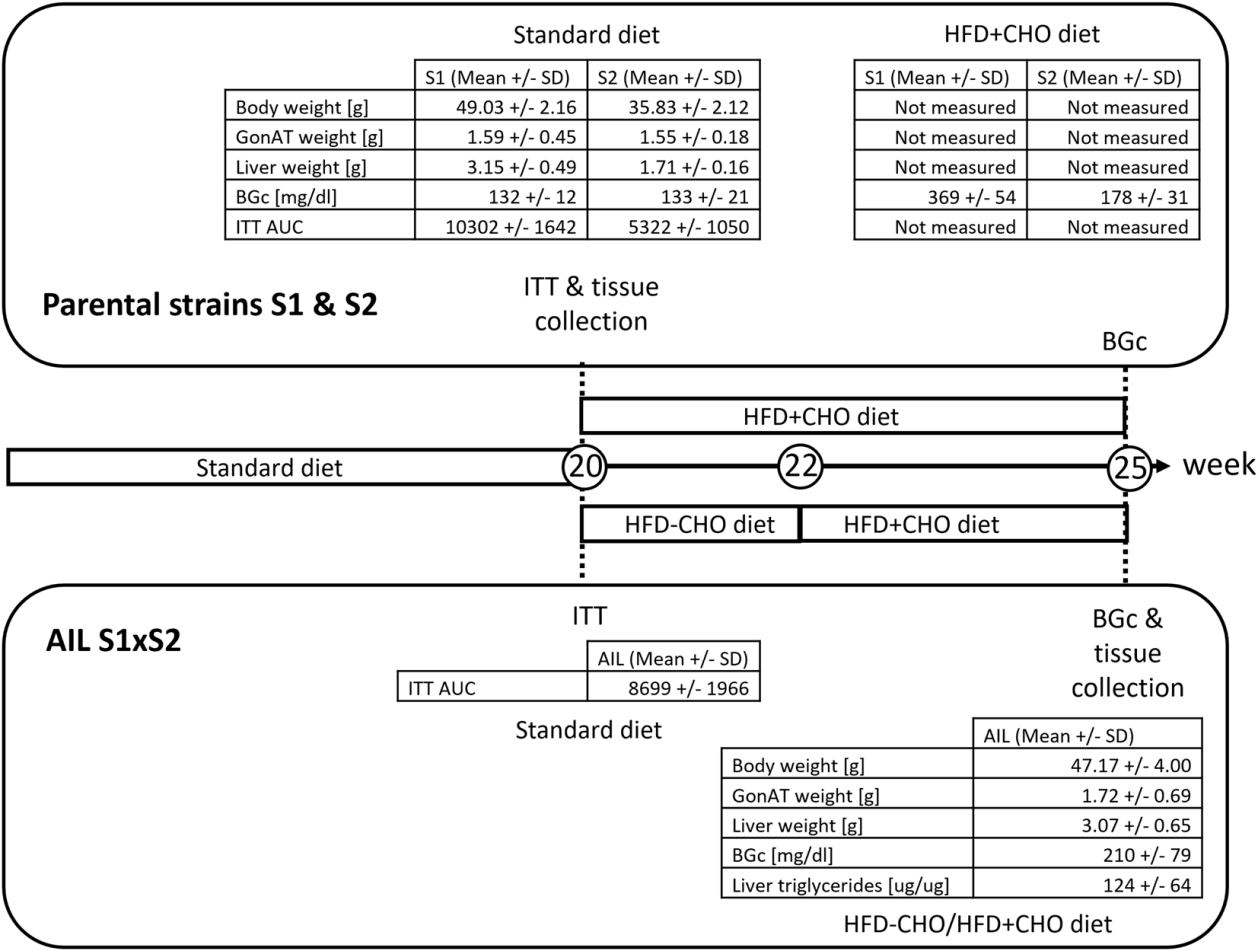
Response of parental lines S1 and S2, and AIL males to high-fat, high-carbohydrate diet. GonAT, gonadal adipose tissue; BGc, blood glucose concentration; ITT, insulin tolerance test; AUC, area under the curve; HFD-CHO, high-fat/low-carbohydrate diet; HFD + CHO, high-fat/high-carbohydrate diet.

According to SNP chip data (GigaMuga), S1 and S2 animals are 96.4% genetically identical (supplementary Fig. 1b). However, with a standard diet S1 males had significantly higher body weight (*p* < 0.001, *n* = 10) and higher liver weight (*p* < 0.001, *n* = 10) compared to S2 males at 20 weeks of age (Fig. 1). To challenge the glucose homeostasis, we fed 20 weeks-old S1 and S2 mice a high-fat, high-carbohydrate diet for 5 weeks and observed extreme high blood glucose concentration in S1 males (369 ± 54 mg/dl) compared to S2 males (178 ± 31 mg/dl) (Fig. 1). To elucidate the genetic impact on the response to this challenge the AIL population was exposed to a gluco-lipotoxic environment provoking differences in β-cell resilience [9]. Therefore, the diet was switched at 20 weeks from a standard diet to a lipotoxic high-fat, low-carbohydrate diet (two weeks) to increase obesity, followed by a gluco-lipotoxic high-fat, high-carbohydrate diet for additional three weeks to challenge β-cells. At 25 weeks AIL mice showed an average blood glucose concentration of 210 ± 79 mg/dl. In addition, gonadal adipose tissue weight was 1.72 ± 0.69 g and liver weight was 3.07 ± 0.65 g on average. Liver triglycerides/protein content was 124 ± 64 μg/μg, and body weight was 47.17 ± 4.00 g on average (Fig. 1).

### Correlation between traits

In metabolically healthy individuals of our AIL we would expect high body weights associated with high adipose tissue weight, unchanged liver weight, and normal glucose clearance. In contrast, the correlation analysis showed no correlation of body weight with gonadal adipose tissue weight (*r* = 0.02, *p* = 0.65), but a positive correlation with liver weight (*r* = 0.65, *p* = 2.20E-16) and liver triglycerides (*r* = 0.39, *p* = 1.94E−15) (Table 1) (Supplementary Fig. 2). Moreover, a negative correlation was found between gonadal adipose tissue and liver weight (*r* = −0.47, *p* = 2.2E-16), and gonadal adipose tissue weight and liver triglycerides (*r* = −0.33, *p* = 2.88E−11). Low adipose tissue weight together with high body weight and high liver weight was also associated with a large area under the curve for the blood glucose concentration in the ITT (*r* = −0.41, *p* = 2.2E−16) and high blood glucose concentration (*r* = −0.59, *p* = 2.2E-16). Consistent with the negative correlation coefficients for adipose tissue weights and the other parameters, positive correlations were found between liver weights and the same parameters (Table 1) (supplementary Fig. 2).

**Table 1:** Correlation coefficients between the collected traits in the AIL population.

	Liver weight	Liver triglycerides/protein	BGc	ITT AUC	Body weight
	(*r* and *P* value)	(*r* and *P* value)	(*r* and *P* value)	(*r* and *P* value)	(*r* and *P* value)
GonAT weight	−0.47, 2.20E-16	−0.33, 2.88E-11	−0.59, 2.20E-16	−0.41, 2.20E-16	0.02, 0.65
Liver weight		0.55, 2.20E-16	0.71, 2.20E-16	0.27, 3.98E-08	0.65, 2.20E-16
Liver triglycerides/protein			0.45, 2.20E-16	0.28, 1.41E-08	0.39, 1.94E-15
BGc				0.34, 9.55E-13	0.31, 2.55E-10
ITT AUC					0.21, 2.56E-05

GonAT, gonadal adipose tissue; BGc, blood glucose concentration; ITT, insulin tolerance test; AUC, area under the curve.

### QTL mapping

QTL mapping was performed for body weight, gonadal adipose tissue weight, liver weight, liver triglycerides, and blood glucose concentration at the end of the experiment and for ITT AUC at 20 weeks before the diet switch. The QTL analysis on selectively genotyped 200 AIL males revealed significant loci on Chr 3, 15, 16, and 17 (supplementary Table 1). The follow-up analysis after KASP genotyping including all 397 males confirmed all four QTL and provided true estimates for the genetic effects (Table 2).

**Table 2:** Position and effects of QTL identified in the AIL population of 397 mice.

Traits	QTL confidence interval					LOD(BH)	Var %	Mean S1	Δ Mean S1-HET	Δ Mean S1-S2
	QTL name	Chr	StartPos	TopPos	StopPos					
GonAT weight [g]	Gatlgq	17	9 483 181	25 258 903	25 391 933	7.3	8.2	1.26	−0.54	−0.61
Liver weight [g]		17	9 483 181	25 258 903	25 391 933	7.5	8.3	3.32	0.26	0.43
BGc [mg/dl]		17	11 934 634	25 258 903	26 054 796	8	9	260	54	73
GonAT weight [g]	Gatq1	3	95 763 020	98 196 163	100 780 367	6.3	6.4	1.49	−0.27	−0.47
BGc [mg/dl]		3	95 763 020	98 196 163	100 543 098	4.2	4.2	194	−13	−44
Body weight [g]	Bwq26	16	3 892 297	11 120 784	21 355 904	7.1	7.2	48.28	1.73	3.15
GonAT weight [g]	Gatq2	15	67 855 285	68 461 862	74 582 319	4.2	4.1	1.49	−0.23	−0.37

GonAT gonadal adipose tissue; BGc, blood glucose concentration; QTL quantitative trait locus; Chr chromosome number; StartPos, TopPos, and StopPos, position of the start of the QTL confidence interval, position of the SNP with the highest LOD score, and position of the end of the QTL confidence interval in base pairs, respectively; Positions are given according to the Mouse Genome Version MM10, GRCm38.p3. SNP, single-nucleotide polymorphism. The confidence interval gives the 1.5 LOD drop region of the top SNP position. A LOD score above 4.9 was deemed to be “genome-wide highly significant” and above 4.2 was deemed “genome-wide significant”; BH, Bonferroni Benjamini–Hochberg correction; LOD, logarithm (base 10) of odds; Var %, percentage of total variance explained; The Δ Mean columns show the phenotypic difference between homozygous S1 and heterozygous BFMI animals (S1-HET) and the difference between homozygous S1 and S2 animals (S1–S2).

**Fig. 2:**
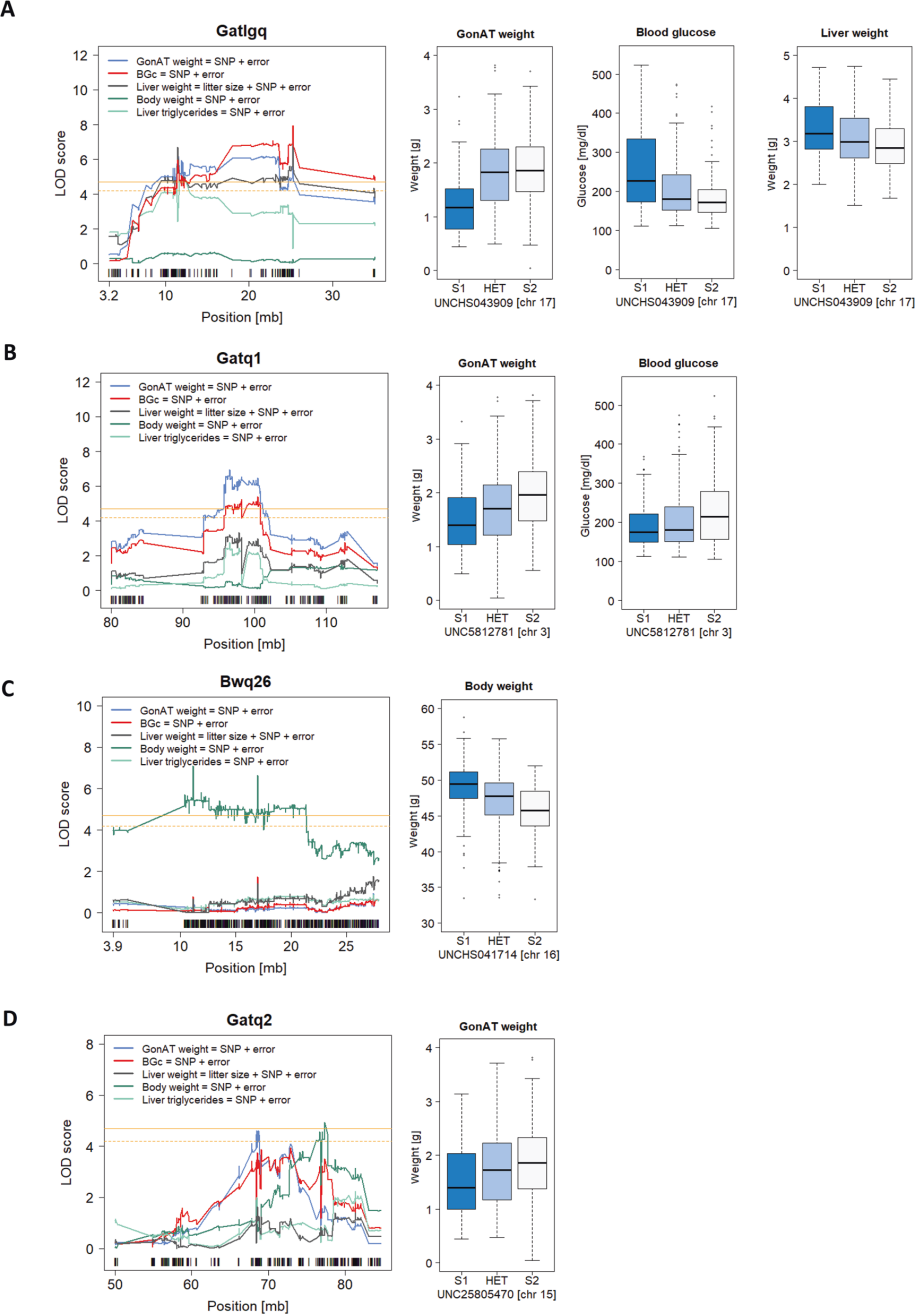
Significant QTL identified in the AIL population. LOD score profiles and effect plots for top SNPs of significant traits (*n* = 397) for **A** Gatlgq, **B** Gatq1, **C** Bwq26, **D** Gatq2. Blue line - gonadal adipose tissue weight, black line – liver weight, red line - blood glucose concentration, dark green line – body weight, light green line – liver triglycerides. QTL, quantitative trait locus; GonAT, gonadal adipose tissue; BGc, blood glucose concentration; SNP, single-nucleotide polymorphism; Chr, chromosome number; HET, heterozygous.

Three significant QTL for gonadal adipose tissue weight were identified on Chr 17 (Gatlgq) at 25.25 Mb (LOD = 7.3), Chr 3 (Gatq1) at 98.19 Mb (LOD = 6.3), and on Chr 15 (Gatq2) at 68.46 Mb (LOD = 4.2). Interestingly, for these three QTL, the S1 allele always decreased the amount of adipose tissue. Gatlgq had also an effect on liver weight (LOD = 7.5) which could be caused by an increased hepatic fat storage. Indeed, liver triglycerides show a significant effect on Gatlgq based on genome-wide multiple testing correction when we consider the selectively genotyping of the initial 200 selected animals (LOD = 4.8). Mapping liver triglycerides using the whole population there is still an effect (LOD = 2.4). However, this effect does not reach the threshold for genome-wide significance (<0.05) but is still suggestive (*P* < 0.1). Furthermore, Gatlgq and Gatq1 affected the blood glucose concentration (LOD Gatlgq = 8, LOD Gatq1 = 4.2). For Gatlgq the allele of the insulin-resistant S1 line was responsible for low adipose tissue weight, elevated liver weight, higher liver triglycerides, and high blood glucose concentration. The S1-allele effects of the Gatlgq on gonadal adipose tissue weight and liver weight and on gonadal adipose tissue weight and liver triglycerides were in opposing direction, supporting the negative correlation between the traits. In contrast, for Gatq1 the S1-QTL allele decreased the adipose tissue weight, reduced the blood glucose concentration (Table 2, Fig. 2b), and was associated with faster glucose clearance in the ITT (LOD = 3.8).

A QTL for body weight was mapped on Chr 16 (Bwq26) at 11.12 Mb (LOD = 7.1). At this locus, the S1 allele was increasing body weight (Table 2).

Since Gatlgq and Gatq1 showed pleiotropic effects on several traits, causal modeling was performed. Causal modeling of Gatlgq suggests direct effects on gonadal adipose tissue, liver weight, and blood glucose concentration. Causal modeling of Gatq1 showed a direct effect of Gatq1 on gonadal adipose tissue weight, which in turn affects blood glucose concentration.

### Candidate gene prioritization

**Fig. 3:**
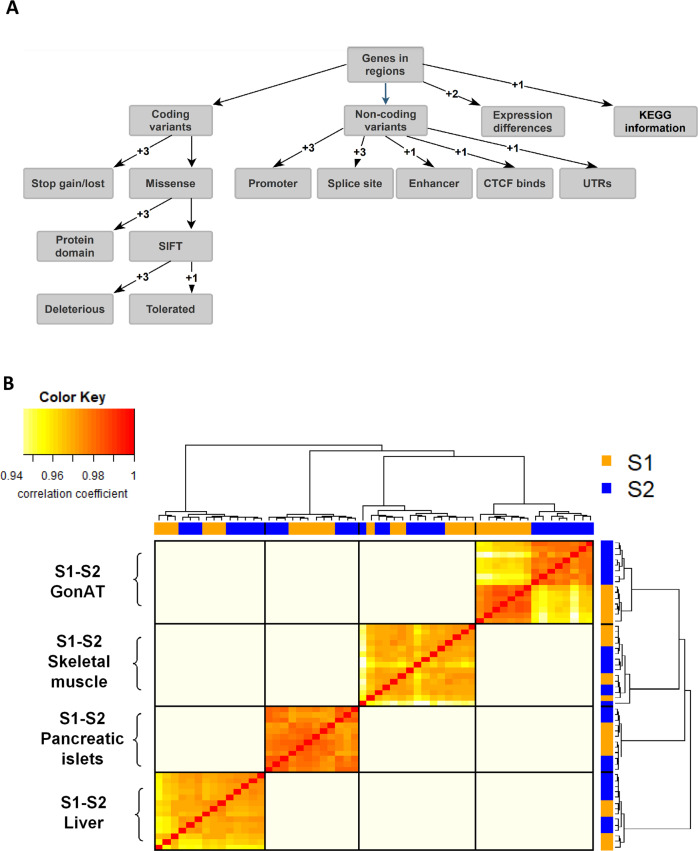
Prioritization of positional candidate genes and identification of the main causal tissue. **A** Decision tree for prioritization of candidate genes located in a QTL region. Genes in a QTL region containing sequence variants between the parental lines S1 and S2 were ranked according to the sum of scores based on the functional annotation of coding and non-coding variants, gene expression data, and KEGG information. **B** Heatmap and dendrogram of microarrays gene expression data from four different tissues (gonadal adipose tissue, skeletal muscle, pancreatic islets and liver) of the parental lines (S1 and S2).

The confidence intervals of the four significant QTL contain 534 protein-coding potential candidate genes. Sixty-two genes were polymorphic between S1 and S2; 27 in Gatlgq, 27 in Gatq1, 4 in Bwq26, 4 in Gatq2. Mutations in these genes were scored for their potential functional effects on the quality or expression level of the encoded protein according to the decision tree (Fig. 3a). None of the genes carried a stop gain or stop loss mutation. Nevertheless, different mutations influencing protein sequence or gene regulation occurred (supplementary Table 2). According to microarrays analysis, considering the 62 candidate genes, we found 37 genes differentially expressed between S1 and S2 in the gonadal adipose tissue, 8 in the liver, 8 in pancreatic islets, and 3 in skeletal muscle (Supplementary Files 5, 6, 7, and 8, respectively). Since correlation analysis of gene expression data between all examined animals showed that mice of the same mouse line clustered together only with gene expression data of the gonadal adipose tissue (Fig. 3b), gonadal adipose tissue is suggested as the main tissue contributing to obesity and glucose homeostasis in the S1 line.

Genes with the highest and second-highest score in every QTL confidence interval were regarded as top candidates (Table 3). Differences in the expression of the top candidate genes for each QTL were confirmed by quantitative real-time PCR in both gonadal adipose tissue and liver (supplementary Fig. 3). *Plg* (plasminogen) and *Acat2* (acetyl-CoA acetyltransferase 2, cytosolic) are the top candidates in Gatlgq. *Plg* was not differentially expressed but contains one tolerated missense variant in the low-complexity region, one SNP in the promoter, and additional SNPs in enhancers and CTCF binding sites in mice of the S1 line. *Acat2* was lower expressed in gonadal adipose tissue of S1 mice (*p* = 7.45E-06) and carries a deleterious missense variant in the thiolase, N-terminal domain. In Gatq1 *Fmo5* (flavin-containing monooxygenase 5) and *Notch2* (notch homolog 2) were prioritized. *Fmo5* was lower expressed in gonadal adipose tissue (*p* = 3.83E-03) and liver (9.11E-08) of S1 versus S2 mice. The Fmo5 gene in S1 mice carries one tolerated missense variant in the FMO-like domain, SNPs in the promoter, and additional SNPs in enhancers and untranslated regions. *Notch2* (*p* = 1.29E-03) was higher expressed in gonadal adipose tissue of S1 mice and carries one deleterious missense variant located in the EGF-like domain plus SNPs in untranslated regions in S1 mice. *Trap1* (TNF receptor-associated protein 1) and *Rrn3 (*RRN3 homolog, RNA polymerase I transcription factor) ranked highest in Bwq26. Both candidate genes *Trap1* (*p* = 5.50E-05) and *Rrn3* (*p* = 2.05E-06) were lower expressed in gonadal adipose tissue, and *Rrn3* was additionally significantly lower expressed in the liver (*p* = 1.28E-06) of S1 mice. Both *Trap1* and *Rrn3* carry one tolerated missense variant. For Gatq2 *Trappc9* (trafficking protein particle complex subunit 9) and *Zfat* (zinc finger and AT hook domain containing) ranged as top candidates. *Trappc9* was lower expressed in S1 *versus* S2 mice in both gonadal adipose tissue (*p* = 5.89E-05) and liver (*p* = 1.92E-04). *Trappc9* possesses variants in UTRs, CTCF binding sites, enhancer, and promoter. *Zfat* was higher expressed (*p* = 2.36E-03) in gonadal adipose tissue of S1 mice and it carries one deleterious missense variant in the low-complexity region in S1 mice.

**Table 3:** Expression differences of prioritized positional candidate genes between males of the parental lines BFMI 861-S1 (*n* = 7) and BFMI861-S2 (*n* = 8). Bold indicates significant differences. The *p* values are corrected according to Benjamini-Hochberg.

QTL name	Chr	Candidate Gene	Type of mutation	FC GonAT (S1/S2)	*P* value GonAT	FC liver (S1/S2)	*P* value liver	Gene score
Gatlgq	17	*Plg*	Tolerated domain missense, CTCF binds, enhancer, and promoter variant	0.12	0.23	0.02	4.66E-03	10
	17	*Acat2*	Deleterious domain missense variant	−0.21	**7.45E−06**	−0.02	0.28	9
Gatq1	3	*Fmo5*	Tolerated domain missense, UTRs, enhancer, and promoter variant	−0.07	**3.83E−03**	−0.1	**9.11E−08**	12
	3	*Notch2*	Deleterious domain missense and UTRs variant	0.05	**1.29E−03**	−0.06	0.02	10
Bwq26	16	*Trap1*	Tolerated domain missense variant	−0.06	**5.50E−05**	−0.01	0.42	7
	16	*Rrn3*	Tolerated missense variant	−0.08	**2.05E−06**	−0.12	**1.28E−06**	4
Gatq2	15	*Trappc9*	UTRs, CTCF binds, enhancer, and promoter variant	−0.06	**5.89E−05**	−0.07	**1.92E−04**	9
	15	*Zfat*	Deleterious domain missense variant	0.05	**2.36E−03**	−0.03	0.25	8

GonAT*,* gonadal adipose tissue; Chr*,* chromosome; FC*,* fold change.

## Discussion and conclusions

To better understand the differences in insulin sensitivity in two sub-lines of the Berlin Fat Mouse independent of a major obesity QTL on Chr 3 (*jObes1*) and to unravel the genetic architecture underlying the observed aspects of the metabolic syndrome we investigated an advanced intercross population of the BFMI861 mouse lines S1 and S2. Besides being genetically closely related and sharing the known obesity locus on Chr3 [5], the two parental mouse lines differ extremely in their metabolic phenotype. The mouse line S1 showed clear features of the metabolic syndrome while S2 was also obese, but had normal glucose homeostasis even under a high-fat, high-carbohydrate diet feeding. The extreme phenotypic data propose the examined mouse lines as an excellent model for studying the genetic determinants of traits of the metabolic syndrome. Due to the random mixture of the genomes of the BFMI861-S1 and -S2 lines, the AIL individuals showed a wide range of phenotypes. Different from expectations in metabolically healthy individuals, we found no correlation between body weight and gonadal adipose tissue weight and negative correlations between gonadal adipose tissue weight and all other traits in AIL males, while liver weight was positively correlated with all other traits. These findings indicate ectopic fat storage in the liver which was indeed confirmed by the assessment of hepatic triglycerides in our AIL. Hepatic fat storage is also present in individuals of the S1 line [6]. Ectopic fat storage in the liver instead of storage in the adipose tissue as the major fat storage organ has been reported repeatedly as causal defect for later impaired glucose clearance [27, 28]. Therefore, we suggest this shift as the likely driver for impaired glucose homeostasis in our mouse model. Gene expression data further supported the assumption of impaired adipose tissue function being causal for the observed phenotypes of the metabolic syndrome in S1 mice. For example, we found distinguished clusters of differentially expressed genes in gonadal adipose tissue between S1 and S2 animals but not in liver, muscle, and pancreatic islets.

The overlap of QTL effects in some regions is consistent with the correlations that we found between the affected traits. For Gatlgq, the S1 allele reduces adipose tissue weight and increases the liver weight and hepatic fat content. Moreover, by shifting fat storage from adipose tissue to ectopic storage in the liver blood glucose concentration is increased. In contrast, the S1 allele on Gatq1 contributes to lower adipose tissue weight, lower blood glucose concentration, and increased insulin sensitivity.

To disentangle the direct and indirect genetic effects of the different QTL, we performed causal modeling. Using causal inference, we were able to provide evidence that out of the two QTL associated with blood glucose concentration in our population, likely only Gatlgq has a direct influence on blood glucose concentration. The second QTL, Gatq1, influences blood glucose concentration indirectly through the regulation of fat storage in adipose tissue, whose weight is directly affected by this QTL. A possible explanation for the discrepancy in the correlation of adipose tissue weight to blood glucose concentration of the two QTL could be that a reduced adipose tissue mass via the S1 allele of Gatlgq could indicate a shift towards ectopic fat storage which is reflected in elevated liver weight and liver triglycerides and thereby contributes to higher blood glucose concentration. In contrast, Gatq1 and Gatq2 could harbor S1 alleles protecting against obesity resulting in lower adipose tissue weight which for Gatq1 is accompanied by lower blood glucose concentration. However, the overall phenotype of the insulin-resistant S1 line appears to be driven mainly by the larger effects of Gatlgq. These findings provide strong evidence for the importance of direct genetic effects on adipose tissue, which indirectly contribute to the etiology of impaired glucose homeostasis.

The AIL population used in this study has the advantage of having a high mapping resolution with respect to the call of positional candidate genes. Because the examined AIL accumulates chromosomal recombination over ten generations, the physical length of the QTL regions is relatively short and thereby the number of positional candidate genes low. In our study, the number of positional candidate genes could be further reduced because long chromosomal stretches are identical between the closely related mouse lines S1 and S2 and, therefore, genes in these regions are not polymorphic and can be excluded from further studies, resulting in 62 out of 534 protein-coding genes as positional candidate genes.

In our prioritization approach, *Plg* and *Acat2* are the top candidate genes for direct effects of Gatlgq on gonadal adipose tissue weight, liver weight, and blood glucose concentration. *Plg* possesses one tolerated missense variant in the low-complexity region of the protein in S1 mice, a region that is significant for the functionality of this protein [29]. *Plg*-knockout mice have lower amounts of adipose tissue [30]. During cell differentiation plasminogen binding is increasing in 3T3 cells and isolated adipocytes suggesting a role in adipose tissue development [31]. In humans, *Plg* was reported to be relevant for the development of insulin resistance and diabetes [32–34]. Thus, *Plg* could be causal for the impaired glucose homeostasis by modifying adipose tissue in S1 mice. *Acat2* is involved in the biosynthesis of fatty acids and cholesterol and is mainly expressed in the liver and intestine [35]. In S1 mice, *Acat2* carries one deleterious mutation that leads to a Valine/Methionine substitution at amino acid position 216 located in the conserved N-terminal domain. This domain is important for the thiolase activity and a mutation in this region could have effects on the protein function. Thus, *Acat2* is a good candidate for the observed hepatic fat storage in S1 mice.

*Fmo5* and *Notch2* were identified as the most likely candidate genes in Gatq1 affecting gonadal adipose tissue weight directly. S1 mice carry two SNPs in the promoter of *Fmo5*. According to the Ensembl database one SNP affects a transcription factor binding site for *Elf5*. The other SNP affects two transcription factor binding sites; one for *Rxra* and one for multiple transcription factors such as *Nr2f6*, *Rara*, *Rarb,* and *Rarg*. According to Bgee database, all identified transcription factors that potentially bind to the promoter region of *Fmo5* are expressed in adipose tissue. The identified SNPs in the promoter of *Fmo5* could therefore be responsible for its lower expression in the gonadal adipose tissue and liver of S1 mice. Consistent with the QTL allele effect of S1 mice leading to lower adipose tissue weight and lower blood glucose concentration, *Fmo5* knockout mice store less fat in gonadal adipose tissue and have lower blood glucose concentration at 20 weeks [36]. *Notch2* is important for developmental processes by controlling cell fate decisions [37] and lipid storage [38]. *Notch2* has been linked to type 2 diabetes in humans [39]. S1 mice carry a deleterious mutation leading to a Glycine/Serine substitution at amino acid position 136. This mutation resides in the EGF-like domain which is important for Notch2 activation [40] and could be causal for the low-fat deposition in gonadal adipose tissue found in S1 mice.

Based on the findings of this genetic study, additional research is necessary to further validate the suggested candidate genes. This could be done by knockout of certain genes, or through continuation of the AIL to reduce the physical length of QTL regions and thereby the number of candidate genes. It is important to note that, although we have prioritized candidate genes using all available information, we cannot completely rule out that one of the polymorphic genes or even an unannotated gene was wrongly discarded.

The human metabolic syndrome is a complex disease with many actors, many still unknown, contributing to its expression. The identification of new potential partners in the network by QTL analysis and subsequent data analysis could help to replenish the gaps.

## Supplementary information

Supplementary Figure 1

Supplementary Figure 2

Supplementary Figure 3

Supplementary Table 1

Supplementary Table 2

Supplementary File 1

Supplementary File 2

Supplementary File 3

Supplementary File 4

Supplementary File 5

Supplementary File 6

Supplementary File 7

Supplementary File 8
