# Supplementary material for: Identification of four novel QTL linked to the metabolic syndrome in the Berlin Fat Mouse
Source: Int J Obes (Lond). 2021 Oct 23;46(2):307–15. doi: 10.1038/s41366-021-00991-3 (PMC8794782; doi:10.1038/s41366-021-00991-3)
Supplement: Supplementary file 1 — Supplementary Figure 1 [file 41366_2021_991_MOESM1_ESM.docx]

**Supplementary Figure 1**


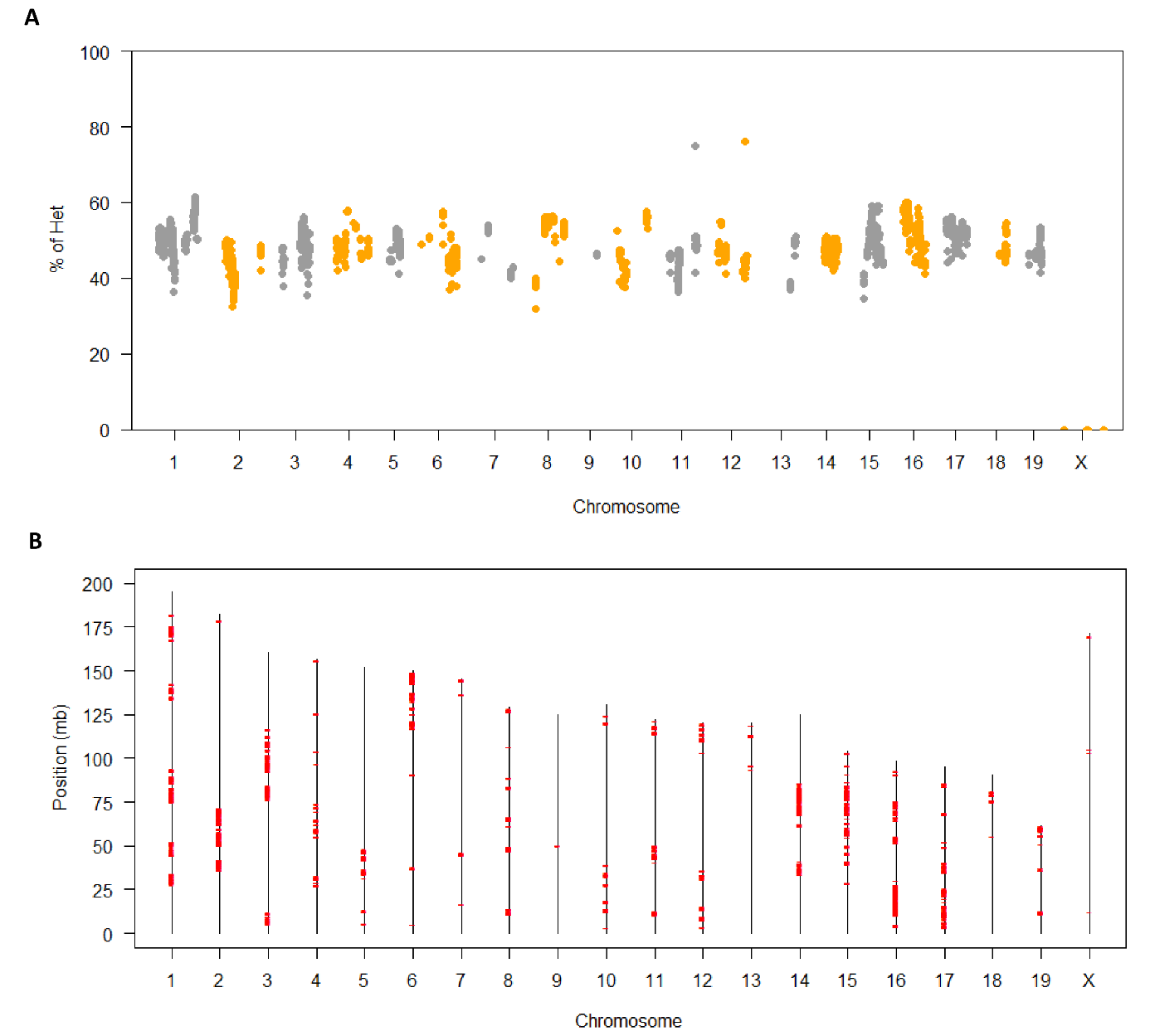


**Figure S1**. (A) Percentage (%) of heterozygosity for each informative SNP (5,215) in the AIL population. (B) Distribution of informative SNPs across the genome in the AIL population.
