# Supplementary figures and images for: Identification of four novel QTL linked to the metabolic syndrome in the Berlin Fat Mouse
Source: Int J Obes (Lond). 2021 Oct 23;46(2):307–15. doi: 10.1038/s41366-021-00991-3 (PMC8794782; doi:10.1038/s41366-021-00991-3)

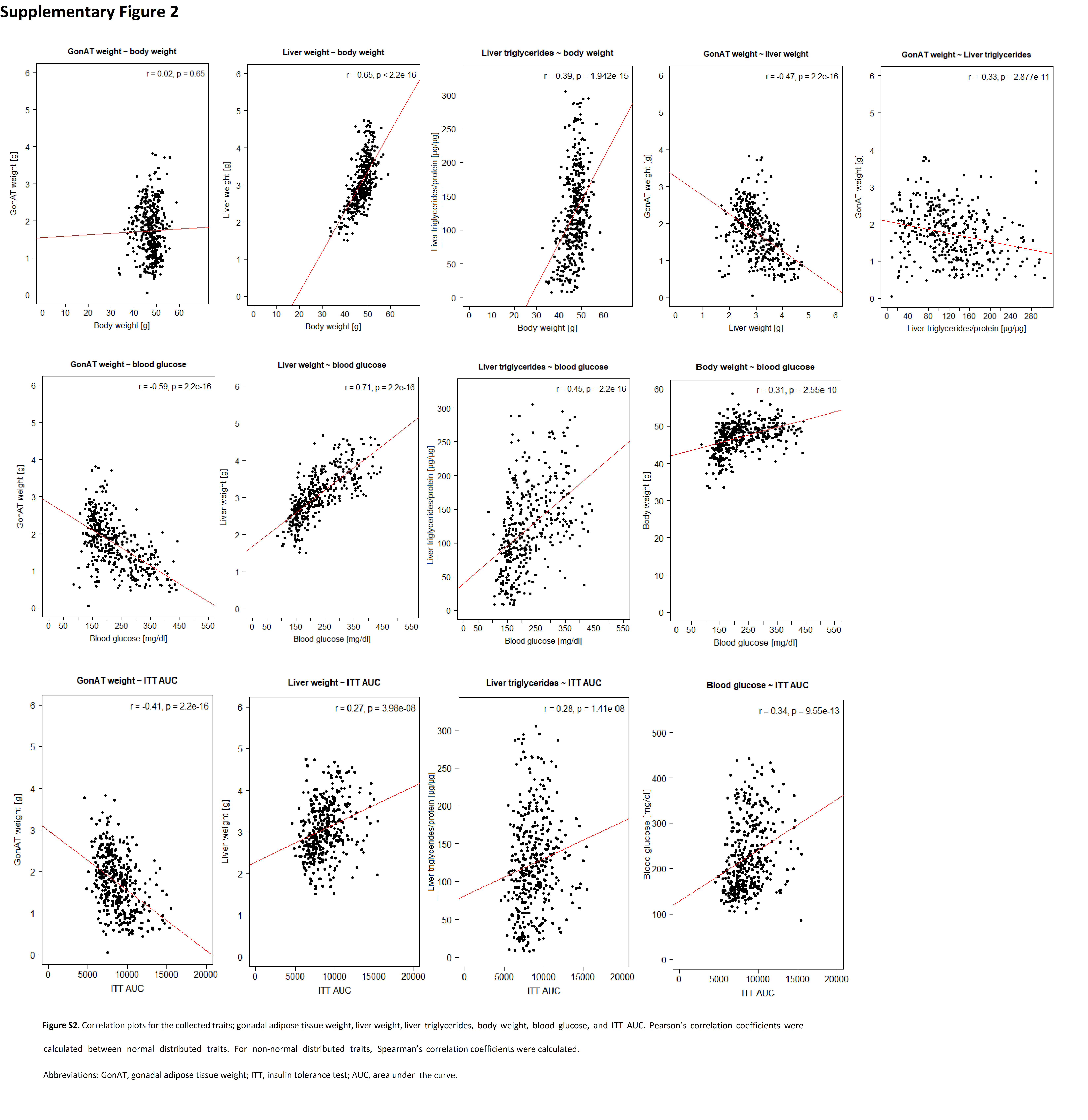

Supplement: Supplementary file 2 — Supplementary Figure 2 [file 41366_2021_991_MOESM2_ESM.png]
