# Supplementary material for: Identification of four novel QTL linked to the metabolic syndrome in the Berlin Fat Mouse
Source: Int J Obes (Lond). 2021 Oct 23;46(2):307–15. doi: 10.1038/s41366-021-00991-3 (PMC8794782; doi:10.1038/s41366-021-00991-3)
Supplement: Supplementary file 3 — Supplementary Figure 3 [file 41366_2021_991_MOESM3_ESM.docx]

**Supplementary Figure 3**

**
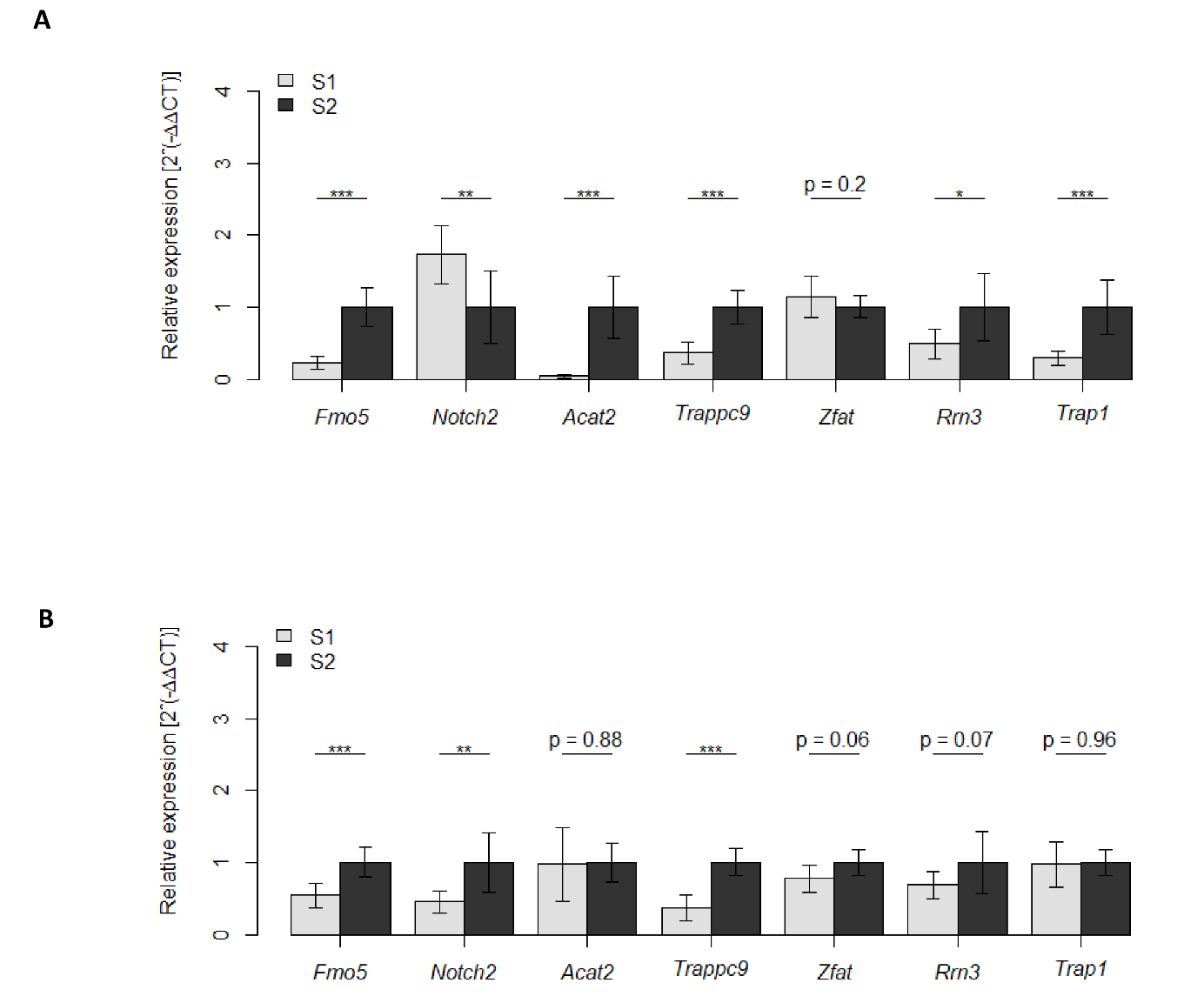
**

**Figure S3**. Validation of microarrays gene expression data for the top candidate genes through semi-quantitative real time PCR in both (A) gonadal adipose tissue (S1: n=7, S2: n=8) and (B) liver (S1: n=7, S2: n=8). Given are transcript amounts in the S1 line relative to S2. Transcript amounts were normalized with the endogenous control *Gapdh*.
