# Supplementary material for: Identification of four novel QTL linked to the metabolic syndrome in the Berlin Fat Mouse
Source: Int J Obes (Lond). 2021 Oct 23;46(2):307–15. doi: 10.1038/s41366-021-00991-3 (PMC8794782; doi:10.1038/s41366-021-00991-3)
Supplement: Supplementary file 4 — Supplementary Table 1 [file 41366_2021_991_MOESM4_ESM.pdf]

Traits	QTL confidence interval					LOD(BH)	Var %	Mean S1	Δ Mean S1-HET	Δ Mean S1-S2
	Chr	StartPos	Top SNP ID	TopPos	StopPos					
GonAT weight [g]	3	95 763 020	UNC5812781	98 196 163	100 780 367	6.5	12.6	1.32	-0.35	-0.89
BGc [mg/dl]	3	95 763 020	UNC5812781	98 196 163	100 543 098	5.2	10	185	-35	-82
GonAT weight [g]	15	67 855 285	UNC25805470	68 461 862	74 582 319	5.1	9	1.29	-0.41	-0.79
Body weight [g]	16	3 892 297	UNCHS041714	11 120 784	21 355 904	5.4	7.2	49.35	2.15	4.06
GonAT weight [g]	17	9 483 181	UNCHS043909	25 258 903	25 391 933	5	11.6	1.14	-0.73	-0.8
Liver weight [g]	17	9 483 181	UNCHS043909	25 258 903	25 391 933	5.5	8.2	3.48	0.41	0.61
BGc [mg/dl]	17	11 934 634	UNCHS043909	25 258 903	26 054 796	6.9	15.1	289	78	105

Abbreviations: GonAT, gonadal adipose tissue; BGc, blood glucose concentration; QTL, quantitative trait locus; Chr, chromosome number; StartPos, TopPos, and StopPos, position of the start of the QTL confidence interval, position of the SNP with the highest LOD score, and position of the end of the QTL confidence interval in basepairs, respectively; Positions are given according to the Mouse Genome Version MM10, GRCm38.p3. Top SNP ID, ID of the top SNP; SNP, single-nucleotide polymorphism. The confidence interval gives the 1.5 LOD drop region of the top SNP position. A LOD score above 4.9 was deemed to be 'genome-wide highly significant' and above 4.2 was deemed 'genome-wide significant'; BH, Benjamini-Hochberg correction; LOD, logarithm (base 10) of odds; Var %, percentage of total variance explained; The Δ Mean columns show the phenotypic difference between homozygous S1 and heterozygous BFMI animals (S1-HET) and the difference between homozygous S1 and S2 animals (S1-S2).
