# Supplementary material for: Identification of four novel QTL linked to the metabolic syndrome in the Berlin Fat Mouse
Source: Int J Obes (Lond). 2021 Oct 23;46(2):307–15. doi: 10.1038/s41366-021-00991-3 (PMC8794782; doi:10.1038/s41366-021-00991-3)
Supplement: Supplementary file 5 — Supplementary Table 2 [file 41366_2021_991_MOESM5_ESM.pdf]

	chromosome	Position	Direct QTL effect	Gene	SIFT deleleterious	SIFT tolerated	Domain	Stop gain/lost	Splice site	UTRs	Promoter	CTCF binds	Enhancer	Expression	Annotation	SCORE
Score points					3	1	3	3	3	1	3	1	1	2	1	
	3	97628804-97655282	Gatq1	Fmo5	0	1	3	0	0	1	3	0	1	2	1	12
	3	98013527-98150361	Gatq1	Notch2	3	0	3	0	0	1	0	0	0	2	1	10
	3	98382461-98753812	Gatq1	Zfp697	3	0	3	0	0	0	3	0	0	0	0	9
	3	97560742-97610203	Gatq1	Chd1l	0	1	3	0	0	0	3	0	0	2	0	9
	3	97689824-97888707	Gatq1	Pde4dip	0	1	3	0	0	1	3	0	1	0	0	9
	3	98709255-98724543	Gatq1	Hsd3b2	3	0	3	0	0	0	0	0	0	2	1	9
	3	96798763-96829351	Gatq1	Cd160	0	1	3	0	0	1	3	0	0	0	0	8
	3	98618634-98630252	Gatq1	Hsd3b5	3	0	3	0	0	1	0	0	0	0	1	8
	3	96596636-96599775	Gatq1	Ankrd34a	3	0	3	0	0	1	0	0	0	0	0	7
	3	96670131-96691032	Gatq1	Ankrd35	0	1	3	0	0	0	3	0	0	0	0	7
	3	95760341-95818863	Gatq1	Rprd2	0	0	0	0	0	1	3	1	1	0	0	6
	3	96172332-96177564	Gatq1	Sf3b4	0	0	0	0	0	1	3	0	0	2	0	6
	3	96904693-97077416	Gatq1	Gja5	0	0	0	0	0	1	0	1	1	2	0	5
	3	97658193-97673812	Gatq1	Prkab2	0	0	0	0	0	1	3	0	0	0	1	5
	3	96601149-96626171	Gatq1	Lix1l	0	0	0	0	0	1	3	0	0	0	0	4
	3	96829284-96870926	Gatq1	Pdzk1	0	0	0	0	0	1	3	0	0	0	0	4
	3	96868281-96905346	Gatq1	Gpr89	0	0	0	0	0	1	3	0	0	0	0	4
	3	98445675-98457126	Gatq1	Gm4450	0	1	3	0	0	0	0	0	0	0	0	4
	3	100451628-100489324	Gatq1	Tent5c	0	0	0	0	0	1	3	0	0	0	0	4
	3	99141068-99239186	Gatq1	Wars2	0	0	0	0	0	1	0	1	1	0	0	3
	3	98222156-98236748	Gatq1	Reg4	0	0	0	0	0	1	0	1	1	0	0	3
	3	96711490-96727628	Gatq1	Polr3c	0	1	0	0	0	0	0	1	1	0	0	3
	3	97158777-97177299	Gatq1	Acp6	0	0	0	0	0	1	0	0	0	2	0	3
	3	95929246-95947390	Gatq1	Anp32e	0	0	0	0	0	1	0	0	0	0	0	1
	3	96162004-96171718	Gatq1	Mtmr11	0	0	0	0	0	1	0	0	0	0	0	1
	3	96913566-96926020	Gatq1	Gja8	0	0	0	0	0	1	0	0	0	0	0	1
	3	97901190-97923276	Gatq1	Sec22b	0	0	0	0	0	1	0	0	0	0	0	1
	15	72589620-73061204	Gatq2	Trappc9	0	0	0	0	0	1	3	1	1	2	1	9
	15	68083764-68258856	Gatq2	Zfat	3	0	3	0	0	0	0	0	0	2	0	8
	15	68928420-69101211	Gatq2	Khdrbs3	0	0	0	0	0	1	3	1	0	2	0	7
	15	71431609-71727838	Gatq2	Fam135b	3	0	3	0	0	1	0	0	0	0	0	7
	16	4039971-4077827	Bwq26	Trap1	0	1	3	0	0	0	0	0	0	2	1	7
	16	13780708-13814839	Bwq26	Rrn3	0	1	0	0	0	0	0	0	0	2	1	4
	16	16983382-17047453	Bwq26	Mapk1	0	0	0	0	0	1	0	0	0	0	1	2
	16	13981699-13986888	Bwq26	Ifitm7	0	0	0	0	0	1	0	0	0	0	0	1
	17	12378608-12419385	Gatlgq	Plg	0	1	3	0	0	0	3	1	1	0	1	10
	17	12942890-12960747	Gatlgq	Acat2	3	0	3	0	0	0	0	0	0	2	1	9
	17	12923813-12940600	Gatlgq	Acat3	3	0	3	0	0	1	0	0	0	0	1	8
	17	25133391-25162104	Gatlgq	Clcn7	0	0	0	0	0	1	3	1	1	2	0	8
	17	25374285-25433783	Gatlgq	Cacna1h	3	0	3	0	0	0	0	0	0	0	1	7
	17	25016085-25099495	Gatlgq	Ift140	0	1	0	0	0	0	3	0	0	2	1	7
	17	25162461-25171913	Gatlgq	Ccdc154	3	0	3	0	0	1	0	0	0	0	0	7
	17	12227621-12318796	Gatlgq	Map3k4	0	1	3	0	0	0	0	1	1	0	0	6
	17	10403012-10840311	Gatlgq	Pacrg	0	0	0	0	0	1	3	0	0	2	0	6
	17	10840384-12063361	Gatlgq	Prkn	0	0	0	0	0	1	3	1	1	0	0	6
	17	15704967-15772610	Gatlgq	Chd1	0	0	0	0	0	1	3	0	0	2	0	6
	17	25120760-25125292	Gatlgq	Ptx4	3	0	3	0	0	0	0	0	0	0	0	6
	17	25188397-25234443	Gatlgq	Unkl	0	0	0	0	0	1	3	1	1	0	0	6
	17	25233331-25240124	Gatlgq	Gnptg	0	0	0	0	0	1	3	0	0	2	0	6
	17	12841079-12868143	Gatlgq	Mas1	0	0	0	0	0	1	3	0	0	0	0	4
	17	12911349-12916345	Gatlgq	Mrpl18	0	0	0	0	0	1	3	0	0	0	0	4
	17	12915701-12925067	Gatlgq	Tcp1	0	0	0	0	0	1	3	0	0	0	0	4
	17	23941672-23945607	Gatlgq	Sbp	0	1	3	0	0	0	0	0	0	0	0	4
	17	25242659-25256364	Gatlgq	Baiap3	0	0	0	0	0	1	3	0	0	0	0	4
	17	25261916-25274622	Gatlgq	Ube2i	0	0	0	0	0	1	3	0	0	0	0	4
	17	13108617-13131810	Gatlgq	Unc93a	0	0	0	0	0	1	0	1	1	0	0	3
	17	25184554-25187662	Gatlgq	BC003965	0	0	0	0	0	1	0	0	0	2	0	3
	17	25343245-25345562	Gatlgq	Tpsab1	0	0	0	0	0	1	0	0	0	2	0	3
	17	13061104-13082481	Gatlgq	Tcp10b	0	0	0	0	0	1	0	0	0	0	0	1
	17	25308646-25311876	Gatlgq	Prss28	0	0	0	0	0	1	0	0	0	0	0	1
	17	25318654-25322684	Gatlgq	Prss29	0	0	0	0	0	1	0	0	0	0	0	1
	17	25366305-25369098	Gatlgq	Tpsb2	0	0	0	0	0	1	0	0	0	0	0	1
