# Supplementary material for: Identification of four novel QTL linked to the metabolic syndrome in the Berlin Fat Mouse
Source: Int J Obes (Lond). 2021 Oct 23;46(2):307–15. doi: 10.1038/s41366-021-00991-3 (PMC8794782; doi:10.1038/s41366-021-00991-3)
Supplement: Supplementary file 7 — Supplementary File 2 [file 41366_2021_991_MOESM7_ESM.pdf]

Name	Species	SNP-Name	Chr.	FAM	VIC/HEX	Assay	Direction	A1-FAM	A2- VIC/HEX	C	Sequence
KM_103	Maus	UNCHS041907	16	C	T	**1**	for	AGACTTACTTTGTAGTCTGGGC	CCTAGACTTACTTTGTAGTCTGGGT	CAGGAGGTAGTAGCACATGCCTTT	TGAGACAGGGTTTCCTTGTGTAATATCCCTGGGTGTCCTAGACTTACTTTGTAGTCTGGG **[T/C]** TGCCTCCCAAAGGCATGTGCTACTACCTCCTGGCCTTTCCTCTACTCTTTTTCCTTTTTT
KM_104	Maus	JAX00063853	15	A	C	**4**	rev	AGAACAATTGTTGGAACCTTGTCAGT	GAACAATTGTTGGAACCTTGTCAGG	CCAAAGGAGGAACTGCCAAGAAGTA	GGTAAGATTAGTCTGCAGAGCCCYTGGCTCCAAAGGAGGAACTGCCAAGAAGTAAAGCTG **[A/C]** CTGACAAGGTTCCAACAATTGTTCTCATGYTGTGTGCCTGYGTCCAGACTTGTAGCACTG
KM_105	Maus	UNCHS043909	17	A	G	**3**	for	CCATCTGGGAGACTTCGGAGA	CCATCTGGGAGACTTCGGAGG	CTGGACAGATGTCAAGAGCTTGTGTT	CCAAGAGCAACACAAGGMATACTATGCGGTTGGTCTGAGTCCATCTGGGAGACTTCGGAG **[G/A]** GTCTGCTGGGGAACACAAGCTCTTGACATCTGTCCAGTGAGGGGTAGTGTGGCAGGGCCT
KM_106	Maus	UNC5812781	3	A	G	**3**	for	ATTTCTCAGGACAATCAAATGGCTCA	TCTCAGGACAATCAAATGGCTCG	AGGGGAGCAGCTGATTTGTGAGAA	GCTTTCATGCAGTGATCAATGGAAAAGTGCATGGTATTTCTCAGGACAATCAAATGGCTC **[A/G]** GACTTCTCACAAATCAGCTGCTCCCCTGTAGAATTTGTCAGCATTGMTTTTAGGCTTCAG
KM_107	Maus	UNC25805470	15	A	G	**3**	rev	CAGGTACTATAAGACCTTGGTAGGT	AGGTACTATAAGACCTTGGTAGGC	CTTCTATCATGGCATTGGCTGTGCTA	GCTAACTTCCAAGGTCTGAATGATACTTCTATCATGGCATTGGCTGTGCTACTGTCTGGA **[A/G]** CCTACCAAGGTCTTATAGTACCTGTTACATTATTATTGTATTGGCAGGGCTCTCTCTCTC
KM_109	Maus	UNCHS041714	16	A	G	**3**	for	AGCCTGGGATACAGAATGAGA	CTAGCCTGGGATACAGAATGAGG	GCCACCCCATCCAGTTTATTCATCTT	GCTCTAAAGGCTAAGGTTGGAAGATTATCAGYTCAAGGCTAGCCTGGGATACAGAATGAG **[A/G]** CCACCTCARAGATTAAGATGAATAAACTGGATGGGGTGGCACAAGCCTTTAATCCCAGCA
KM_111	Maus	UNC27568354	17	A	G	**3**	for	CACTTTTGAGGGAACTGCTTCACA	ACTTTTGAGGGAACTGCTTCACG	ATGAGGGAAAAAGGTATCCTCATCTGTAA	TATATGAAAAACATCCTCAATGTTCCTGCCAGCTCAGCACTTTTGAGGGAACTGCTTCAC **[G/A]** TTACAGATGAGGATACCTTTTTCCCTCATGCCTGCAAAGGCAGTTGAAGGCAACCCAGTC
