# Supplementary material for: Identification of four novel QTL linked to the metabolic syndrome in the Berlin Fat Mouse
Source: Int J Obes (Lond). 2021 Oct 23;46(2):307–15. doi: 10.1038/s41366-021-00991-3 (PMC8794782; doi:10.1038/s41366-021-00991-3)
Supplement: Supplementary file 8 — Supplementary File 3 [file 41366_2021_991_MOESM8_ESM.docx]

Causal modeling for GonAT weight and blood glucose concentration was performed on QTL2_gonAT/Glu_ by comparing the following causal models:

1. residuals(Gluc ~ GonAT weight) ~ marker + error
2. residuals(GonAT weight ~ Gluc) ~ marker + error

Causal modeling for GonAT weight, blood glucose concentration and liver weight was performed on QTL1_gonAT/Liv/Glu_ by comparing the following causal models:

Causal modeling 1, liver weight and blood glucose concentration:

1. residuals(Gluc ~ liver weight) ~ marker + error
2. residuals(liver weight ~ Gluc) ~ marker + error

Causal modeling 2, GonAT weight and blood glucose concentration:

1. residuals(Gluc ~ GonAT weight) ~ marker + error
2. residuals(GonAT weight ~ Gluc) ~ marker + error

Causal modeling 3, GonAT weight and liver weight:

1. residuals(liver weight ~ GonAT weight) ~ marker + error
2. residuals(GonAT weight ~ liver weight) ~ marker + error
