# Supplementary material for: Identification of four novel QTL linked to the metabolic syndrome in the Berlin Fat Mouse
Source: Int J Obes (Lond). 2021 Oct 23;46(2):307–15. doi: 10.1038/s41366-021-00991-3 (PMC8794782; doi:10.1038/s41366-021-00991-3)
Supplement: Supplementary file 9 — Supplementary File 4 [file 41366_2021_991_MOESM9_ESM.pdf]

Long original name	Sequence	Length	Modification	Annealing	DNA	Species	Chr.	Gene	Method
		bp		T in °C	type				
Fmo5-fwd	ACTCAAGTGTTCAAAGGGCTA	21	no	57	cDNA(mouse)	Mouse	3	Fmo5	qpcr
Fmo5-rev	GGCCCCAGTAGTAACCGTAA	20		58			3	Fmo5	qpcr
Notch2-fwd	AACATCGAGACCCCTGTGAG	20		59			3	Notch2	qpcr
Notch2-rev	CACTGACACTGCTTCCCTGT	20		59			3	Notch2	qpcr
Acat2-fwd	ATGTCTTGACGGCAGGTTGT	20		59			17	Acat2	qpcr
Acat2-rev	TTAGCTATTGCCGCAGACACC	21		60			17	Acat2	qpcr
Trappc9-fwd	ATTCAGTGGAGAAGCGCATC	20		58			15	Trappc9	qpcr
Trappc9-rev	CCGCTTCTTGTAGTGTCTGC	20		58			15	Trappc9	qpcr
zfat-fwd	AAGAAGCCCAGCACTGAAGA	20		59			15	Zfat	qpcr
zfat-rev	ACTCGTTACCCAGATTCAGCA	21		59			15	Zfat	qpcr
Rrn3-fwd	CATGTCACAGAGCCTTGCAA	20		58			16	Rrn3	qpcr
Rrn3-rev	GCCGCGATACACTCACATC	19		58			16	Rrn3	qpcr
Trap1-fwd	TACAGTCCAGTTTAGGGGCC	20		58			16	Trap1	qpcr
Trap1-rev	AGCTCTCGTATGAACACCTCT	21		58			16	Trap1	qpcr
